# The origin and early evolution of vascular plant shoots and leaves

**DOI:** 10.1098/rstb.2016.0496

**Published:** 2017-12-18

**Authors:** C. Jill Harrison, Jennifer L. Morris

**Affiliations:** 1 School of Biological Sciences, University of Bristol, 24 Tyndall Avenue, Bristol BS8 1TQ, UK; 2 School of Earth Sciences, University of Bristol, 24 Tyndall Avenue, Bristol BS8 1TQ, UK

**Keywords:** land plant, shoot, leaf evolution, evo–devo, plant body plan

## Abstract

The morphology of plant fossils from the Rhynie chert has generated longstanding questions about vascular plant shoot and leaf evolution, for instance, which morphologies were ancestral within land plants, when did vascular plants first arise and did leaves have multiple evolutionary origins? Recent advances combining insights from molecular phylogeny, palaeobotany and evo–devo research address these questions and suggest the sequence of morphological innovation during vascular plant shoot and leaf evolution. The evidence pinpoints testable developmental and genetic hypotheses relating to the origin of branching and indeterminate shoot architectures prior to the evolution of leaves, and demonstrates underestimation of polyphyly in the evolution of leaves from branching forms in ‘telome theory’ hypotheses of leaf evolution. This review discusses fossil, developmental and genetic evidence relating to the evolution of vascular plant shoots and leaves in a phylogenetic framework.

This article is part of a discussion meeting issue ‘The Rhynie cherts: our earliest terrestrial ecosystem revisited’.

## 1. Introduction

Today's biota includes *ca* 375 000 species of vascular plant that generate over 90% of terrestrial productivity, and variation in shoot and leaf form are major components of vascular plant biodiversity [1–3]. The earliest land plants arose about 470 million years ago and are evidenced in the fossil record as spores or spore masses [4–7]. Speculatively, these plants lacked shoots and leaves, instead having tiny fertile axes that entered reproductive development straight away or elaborated a small axis terminating in sporangium formation [8–10], and similar forms remain evident among living bryophyte relatives of the earliest land plants, which comprise *ca* 20 000 species [1]. Around 430 million years ago [11,12], the innovation of shoots and leaves underpinned an explosive radiation of vascular plant form analogous to the Cambrian explosion of animals. The origin of vascular plants precipitated a 10-fold increase in plant species numbers [1], promoted soil development [13] and led to an 8–20-fold atmospheric CO_2_ drawdown [5,14], significantly shaping Earth's geosphere and biosphere [15–17]. Many pro-vascular and early vascular plant forms in the fossil record look very different to modern vascular plants and exhibit traits that suggest stepwise changes in form from a bryophyte-like evolutionary starting point [9–11,18]. Unlike vascular plants, bryophytes have gametophyte-dominant life cycles in which the photosynthetic body of the plant is haploid; vascular plant shoots and leaves evolved in the diploid sporophyte phase of the life cycle [19]. In this review, we aim to give an overview of the stages in vascular plant shoot and leaf evolution evident in the fossil record, explain how developmental and genetic findings in bryophytes and non-seed vascular plants illuminate these steps and identify future research avenues that will tell us more about how vascular plant shoots and leaves arose. The origin of vascular plants with shoots and leaves has intrigued biologists for over 100 years, e.g. [19,20], and the new tools and fossil evidence that we have at our disposal offer the possibility to generate knowledge that will fundamentally advance our understanding of vascular plant form and evolution [10,21–23].

## 2. Identifying the direction of evolutionary trait change

To understand the evolution of plant form, we need to know which traits have been gained or lost through time in the plant lineages that concern us. This aim can be fully realized in studying closely related plants where divergence times are recent and traits of interest are distributed among taxa whose evolutionary relationships are well resolved. For instance, archaeology, dated molecular phylogenies and developmental genetics all support strong branch suppression in the monophyletic origin of maize from its wild relative teosinte around 9000 years ago [24–27]. However, the lineage divergence times involved in leaf evolution are ancient, spanning a period of around 440 million years [11]. Comprehensive sampling of the fossil record is not possible owing to incomplete deposition and taphonomic degradation, and extinct taxa are not open to experimentation in the way that living plants are. These features make it hard to identify the direction of trait change involved in vascular plant shoot and leaf evolution. Nevertheless, a combination of phylogenetic and fossil data illuminates some of the steps involved in the evolution of leafy forms, and these are outlined below.

## 3. Morphological transitions during the origin of vascular plant shoot systems

**Figure 1.**
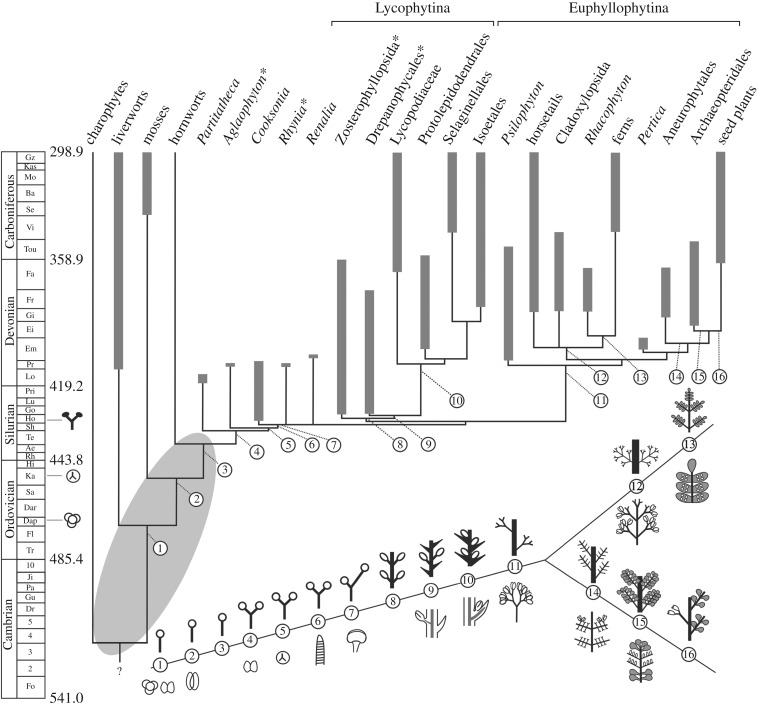
Hypothetical phylogenetic tree for land plants plotted against time in the Palaeozoic, based on the stratigraphic ranges of key taxa and major groups of land plants from the fossil record (thick grey bars) with minimum implied range extensions (thin lines) (modified after Kenrick & Crane [11,31]). Starred taxa or groups were present in the Rhynie chert assemblage. The first appearances of permanent, regularly arranged cryptospores, trilete monads and an unequivocal embryophyte body are indicated against the time-scale. The timing of divergence and inter-relationships between the bryophyte and tracheophyte lineages are not yet resolved so relationships within the grey oval are uncertain; here we follow Kenrick & Crane [11,31]. The maximum age for the origin of the embryophytes is estimated around the mid Ordovician based on the first appearance of tetrahedral cryptospore tetrads [32]. Numbered illustrations indicate the phylogenetic position of key innovations in plant form, with a focus on shoot and leaf evolution. Innovations included 1–3: uni-axial, leafless sporophyte forms (see also figure 2*a–d*), 1: permanent tetrads and dyads, similar to those produced by some extant liverworts [32], 2: stomata (stomatophytes), 4–6: isotomous branching, 4: cryptospores, 5: trilete monads, 6: vascular tissue (tracheophytes), 7: increased branching complexity (anisotomy), 8–10: indeterminate growth with lateral insertion of bivalved sporangia, 9: non-vascularized enations, 10: vascularized lycophylls and positioning of sporangia behind leaves, 11: simple lateral branching systems with sporangia arranged in trusses, 12: complex lateral branching systems with dichotomies lateral to first or second order branches, 13: planar fronds with laminae (in grey) and sporangia positioned on abaxial surfaces, 14: increased complexity in lateral branching systems with dichotomies lateral to first or second order branches and terminal sporangia, 15: planar euphylls on lateral branching systems (in grey) with sporangia positioned on adaxial surfaces, 16: seeds arising on lateral branches.

**Figure 2.**
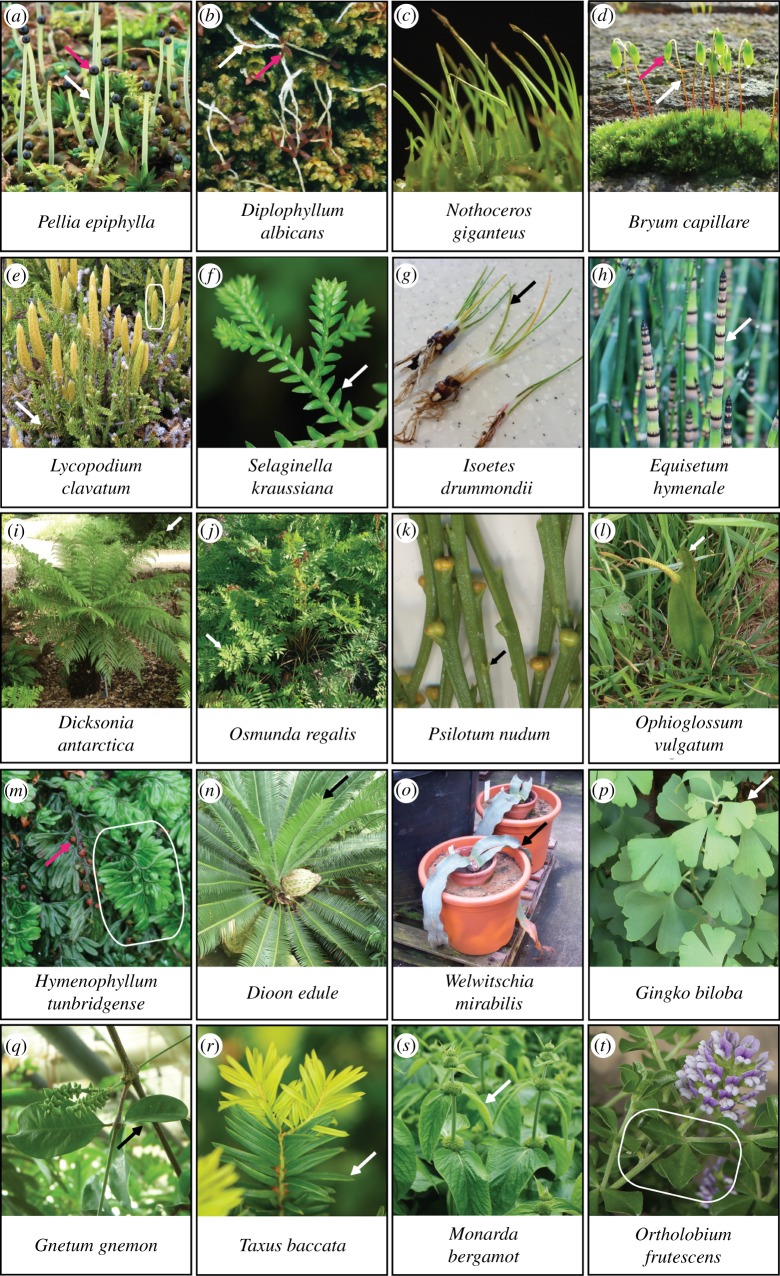
The range of shoot and leaf morphologies among major clades of living land plants (images not to scale). (*a–d*) Thalloid liverwort (*a*), leafy liverwort (*b*), hornwort (*c*) and moss (*d*) sporophyte forms are somewhat similar, comprising a single axis (white arrows) that terminates in sporangium formation and capsule development (pink arrows). Hornwort sporangia run most of the length of the sporophyte and are not labelled. While liverwort sporophytes are fully dependent on gametophytes for food, moss and hornwort sporophytes contain some photosynthetic tissues. (*e–g*) Clubmosses (*e*), spike mosses (*f*) and quillworts (*g*) derive from deep divergences within the lycophyte lineage as outlined in figure 1, and have lycophylls. Sporangia are borne laterally on specialized reproductive shoots termed strobili, as framed in (*e*). (*h–m*) Living monilophytes comprise horsetails (*h*), polypod ferns (*i*,*j*), whisk ferns (*k*), ophioglossid ferns (*l*) and filmy ferns (*m*), which have diverse leaf morphologies reflecting different patterns of development. White or black arrows and the frame in (*m*) indicate leaves or fronds, and pink arrow indicates sporangium. (*n–r*) A selection of leaf morphologies represented among gymnosperms. The familiar pine needle leaf form of conifers represents a narrow aspect of gymnosperm leaf morphology. (*s*,*t*) Simple and compound flowering plant leaves. Photographs contributed by (*a*,*b*) David Long, (*c*,*d*,*e*,*m*) Jeff Duckett and Silvia Pressel, (*f–l*,*n–t*) Jill Harrison and (*g*) Joshua Mylne.

**Figure 3.**
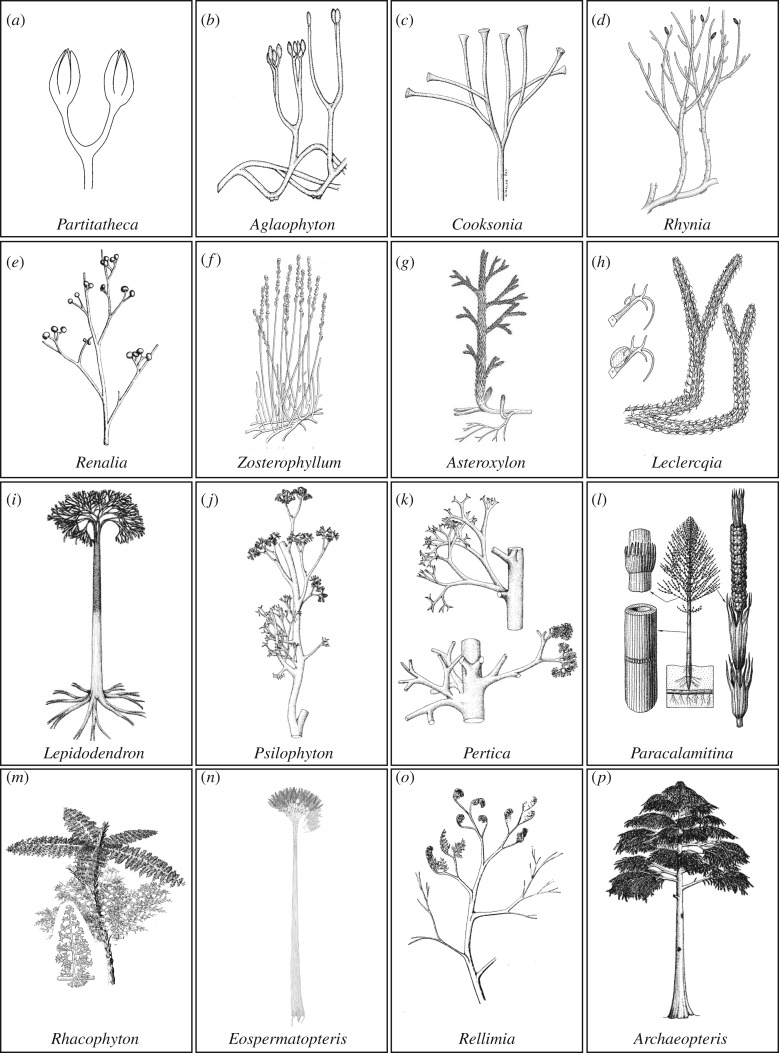
(*Overleaf*.) Shoot system architectures of fossil pro-vascular and vascular plant lineages included in figure 1. These fossils illustrate evolutionary transitions contributing to polyphyletic leaf origins including bifurcation, sterilization, indeterminacy, overtopping, planation and webbing. (*a*,*b*) *Partitatheca* (*a*) and *Aglaophyton* (*b*) represent part of an early pro-vascular or vascular plant grade with bifurcating shoot systems and terminal sporangia. (*c–e*) Among basal vascular plants, increases in shoot size (*Cooksonia*) and developmental complexity are evidenced by sterile and reproductive branch fate acquisition (*Rhynia*) or unequal branch growth to produce an overtopped form (*Renalia*). (*f–i*) Fossil lycophytes have sterile indeterminate axes with lateral sporangia (*Zosterophyllum*) or lycophylls (*g–i*). Branching is isotomous (*h*,*i*) or overtopping (*f*,*g*), and some fossil isoetaleans such as *Lepidodendron* attained tree forms more than 30 m tall. (*j*,*k*) Stem group euphyllophytes such as *Psilophyton* and *Pertica* had overtopped shoot systems with bifurcating lateral branches that were sterile or terminated in sporangial clusters. (*l–n*) Monilophyte fossils include horsetail-like sphenopsids such as *Paracalamitina* (*l*), in which leaves were iterated in whorls, and sporangia differentiated from modules of the main axis or branches. Fern-like plants such as *Rhacophyton* (*m*) had partially planar lateral branches with multiple branchlets and some webbing at the distal tips. Cladoxylopsids such as *Eospermatopteris* (*n*) had a tree-like habit with terminal clusters of flattened lateral branches and multiple dichotomizing branchlets. (*o,p*) Progymnosperms such as *Rellimia* (*o*) and *Archaeopteris* (*p*) had planar lateral branches with multiple branchlets, and in some instances laminar tissue. Reconstructions were (*a*) drawn by Jennifer Morris, (*b*) redrawn from Edwards [34] and reproduced from Edwards [18] by permission of the Royal Society of Edinburgh, (*c*) reproduced from Gerrienne *et al*. [35] by permission of Elsevier, (*d*) redrawn from Edwards [36] and reproduced from Kenrick & Crane [11] with permission from Paul Kenrick, (*e*) redrawn from Gensel [37] and reproduced from Stewart & Rothwell [38] with permission from Cambridge University Press, (*f*) reproduced from Walton [39] with permission from the International Society of Plant Morphologists, (*g*) reproduced from Edwards [18] by permission of the Royal Society of Edinburgh, (*h*) reproduced from Bonamo *et al.* [40] by permission of the Botanical Gazette, (*i*–*k*) reproduced from Stewart & Rothwell [38] with permission from Cambridge University Press, (*l*) reproduced from Naugolnykh [41] with permission from Cambridge University Press, (*m*) reproduced from Stewart & Rothwell [38] with permission from Cambridge University Press, (*n*) reproduced from Stein *et al*. [42] with permission from Nature Publishing Group, (*o*) reproduced from Bonamo & Banks [43] with permission from the Botanical Society of America, (*p*) reproduced from Stewart & Rothwell [38] with permission from Cambridge University Press.

**Figure 4.**
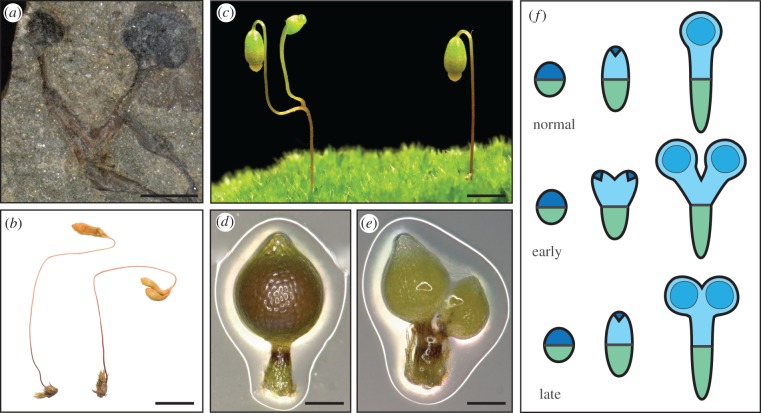
Origins of a polysporangiophyte habit. (*a*) A pro-tracheophyte fossil *Cooksonia* spp. sporophyte. Scale bar, 1.8 mm. (*b*) A rare natural variant of *Bryum radiculosum* showing duplicated sporangia subtended by a portion of seta (photo by Alison Reed reproduced from Edwards & Kenrick [5]). Scale bar, 5 mm. (*c*) A rare natural moss variant showing sessile duplicated sporangia, as described in the classical literature [20,46] (photo by Alison Reed). Scale bar, 5 mm. (*d*) Wild-type sporophyte morphology in the moss *Physcomitrella*. Scale bar, 0.2 mm. Reproduced from Bennett *et al*. [47]. (*e*) *Physcomitrella pinb* mutants have a low penetrance branching phenotype. Scale bar, 0.2 mm. Reproduced from Bennett *et al*. [47]. (*f*) Embryonic development in *Physcomitrella* involves a transverse division to form apical (blue) and basal (green) identities. Apical (dark blue) and basal cells iterate the embryonic axis, and this embryonic development is followed by sporangium differentiation (blue circles) and intercalary proliferation. Speculatively, the branching morphologies of (*b*) and (*c*) involve early and mid-stage division and duplication of the apical cell, respectively. *pin* mutations in a moss-like sporophyte provide one possible entry point into the evolution of polysporangiophyte forms.

Phylogenetic evidence places bryophytes as a monophyletic sister group or paraphyletic sister grade to the vascular plants [28–30], and bryophytes all have uni-axial sporophytes terminating in reproductive sporangium formation (morphologies 1–3 in figures 1 and 2*a–d*), an ancestral characteristic of land plants [10,33]. The first step in shoot evolution involved the innovation of a branching habit with sporangia at the tips of each branch (morphology 4 in figure 1). *Partitatheca* is among the earliest branching fossils. It has small axes (*ca* 3 mm tall) that possess a combination of bryophyte and tracheophyte characters, including an apparent lack of vasculature, production of dyad spores, stomata and branching axes with at least one dichotomy (figures 1 and 3*a*) [5,9,44,45]. *Aglaophyton* (morphology 5 in figures 1 and 3*b*) shows similar composite features with no vasculature, production of trilete monad spores and a higher order of branching [31]. *Cooksonia* fossils (morphology 6 in figure 1; figures 3*c* and 4*a*) exemplify the earliest known vascular plants, and range in height from 1.8 mm to 6 cm [5,48–50]. Some *Cooksonia* fossils have axes that are considered too narrow to contain much photosynthetic tissue and, as in bryophytes, their sporophytes were most likely to have been nutritionally dependent on photosynthetic gametophytes [51]. Their repeated equally branching habit with each branch terminating in sporangium formation (figure 3*c*) suggests repetition of a developmental module that pre-existed in bryophytes and pro-vascular plants such as *Partitatheca*. Similar isotomously branching forms with terminal sporangia are manifest among vascular plants of the Rhynie chert assemblage [18], suggesting that this developmental module was a plesiomorphy of early vascular plants and their precursors (figure 1). Therefore, the earliest vascular plants had a system of equally branching axes with terminal sporangia but no leaves, and such forms are known as polysporangiophytes.

## 4. Stage 1. The origin of bifurcating forms

### (a) Patterns of development in bryophyte sporophytes

The nature of morphological, developmental and genetic change generating polysporangiophyte branching forms has been a source of scientific speculation for over a century, and still remains an open question [5,10,11,20,30,46,49,52–60]. It is now widely accepted that polysporangiophyte forms arose from uni-axial bryophyte-like precursors [5,56–58,60]. However, the uni-axial form of liverworts, mosses and hornworts (figure 2*a–d*) arises by distinct embryonic trajectories both within and between lineages (table 1) [33,57]. In brief, liverwort and moss zygotes undergo a first division to form apical and basal cells, and with the exception of *Riccia*, the sporangium differentiates from the apical end of the embryo [33]. Axial development occurs by apical differentiation into the foot and seta in liverworts or by distinct apical cell and intercalary proliferative activities and differentiation in mosses [33]. Hornwort sporophytes show a divergent pattern of development in which the first embryonic division is vertical and subsequent transverse divisions pattern the embryo. The basal cells arising by transverse divisions differentiate into a foot region, an intercalary proliferative region and a short seta [33]. The bifurcating architectures of early polysporangiophytes are thought to reflect the activity of apical meristems with a single apical stem cell [57,58], and the transient embryonic apical cell activity of mosses may offer the closest living proxy.

**Table 1.** Patterns of embryonic development in bryophytes. Thallose liverworts (TL), leafy liverworts (LL), hornworts (H) and mosses (M); data collated from Parihar [33].

order	species	form	first embryonic division	second embryonic division	growth by apical cell	origin of sporangium	axial elongation
TL	*Riccia crystallina*	globoid	transverse	vertical	absent	apical + basal differentiation	none
TL	*Marchantia polymorpha*	axial	transverse	vertical	absent	apical differentiation	basal differentiation into foot and seta
TL	*Pellia epiphylla*	axial	transverse	vertical	absent	apical differentiation	apical differentiation into foot and seta
LL	*Porella bolanderi*	axial	transverse	transverse	absent	apical differentiation	apical differentiation into foot and seta
LL	*Frullania dilatata*	axial	transverse	transverse	absent	apical differentiation	basal differentiation into foot, apical differentiation into seta
H	*Anthoceros* sp.	axial	vertical	transverse	absent	apical differentiation	basal differentiation into foot, apical differentiation into intercalary meristem and seta
M	*Sphagnum subsecundum*	axial	transverse	transverse	some	apical cell division and differentiation	basal cell divisions and differentiation into foot, no seta elongation
M	*Andreaea* sp.	axial	transverse	oblique	present	apical cell division and differentiation	basal cell divisions and differentiation into foot, no seta elongation
M	*Funaria hygrometrica*	axial	transverse	oblique	present	apical cell division and differentiation	basal cell divisions and differentiation into foot and lower part of seta, apical differentiation into intercalary meristem
M	*Physcomitrella patens*	axial	transverse	oblique	present	apical cell division and differentiation	basal cell divisions and differentiation into foot and lower part of seta, apical differentiation into intercalary meristem

### (b) A note on bryophyte phylogeny and trait change inference

The extent to which inferences from mosses are transferable up the plant tree of life is unclear owing to variability in developmental patterns and currently unresolved phylogenetic relationships among bryophytes. While morphological phylogenies resolve mosses as the sister lineage to vascular plants (implying homology between mosses and early polysporangiophytes), molecular phylogenies are inconclusive or imply non-homology (reviewed in [10]). Growing support for the latter scenario will necessitate identification of developmental mechanisms that are shared among bryophytes as well as among vascular plants to understand the developmental transitions occurring as polysporangiophytes arose.

### (c) Developmental innovations during polysporangiophyte evolution

The patterns of axial development among early polysporangiophytes remain speculative. Some authors have proposed that the bryophyte seta is homologous to the axes of polysporangiophyte forms [57,58] and others have argued that while the bryophyte seta arises from sporangial tissues, a distinct, well-established apical meristem generated early polysporangiophyte forms [52]. Rare natural liverwort and moss variants have branching sporophytes (figure 4), and such variants have received attention in light of hypotheses of polysporangiophyte evolution as they demonstrate that bryophytes can branch, and provide a potential entry point into the evolution of the polysporangiophyte habit [5,20,46]. In mosses, some variants undergo sporangial duplication (figure 4*b*) while others undergo a more extensive apical duplication to produce two sporangia subtended by a portion of seta (figure 4*c–e*) and both of these patterns are represented in the fossil record [5,20,46,61]. Speculatively these variants arise by early or later division of an embryonic apical cell, with an early duplication preceding intercalary proliferative activity and later duplication succeeding intercalary proliferation (figure 4*f*), and the latter form is similar to the form of early polysporangiophyte fossils.

### (d) Experimental evidence for the origin of polysporangiophytes

Reverse genetic data are starting to pinpoint genes that may have been involved in the evolution of polysporangiophyte apical meristem functions. In *Arabidopsis*, *PIN* and *TCP* genes regulate branch initiation [62,63] and suppression of axillary bud activity [64,65] to determine plants' overall branching form. PIN-mediated polar auxin transport is conserved between *Arabidopsis* and moss sporophytes [66], and disruption of PIN function in a moss induces at low penetrance a branching form that closely resembles early polysporangiophyte fossils (figure 4) [47,60] and *PpTCP5* disruption similarly induces branching [67]. Disrupting the function of two other gene classes in *Physcomitrella* can also induce sporophyte branching. *Pplfy* mutants have defective early embryonic divisions that impede sporophyte development, but in rare instances sporophytes are able to develop and they are branched [68]. However, in *Arabidopsis*, LEAFY activates the reproductive transition, and gene pathways for floral development [69], and LEAFY and PpLFY have divergent DNA binding capacities [70]. There are no *PpLFY* gain-of-function mutants and the downstream targets of PpLFY are not yet known, so it is hard to interpret the *Physcomitrella Pplfy* mutant phenotype in light of the evolution of branching. Similarly the low penetrance branching mutant phenotype of *Pptel* mutants is hard to interpret because *TEL* encodes an RNA binding protein, and the specificity of *PpTEL* action is not known [71]. The cellular and developmental basis of branching in the mutants above remains an open question, but the low penetrance of branching phenotypes suggests that an element of stochasticity is involved in the development of moss sporophyte branching, potentially in early embryonic cell fate specification.

## 5. Stage 2. The origin of indeterminate forms

### (a) Patterns of axial development in early vascular plants

Early divergences in the vascular plant lineage gave rise to indeterminate forms with lateral sporangia or sporangia on simple lateral branch systems (figures 1 and 3*d–f*) [31,72]. Thus, a second step in the evolution of shoots with leaves involved displacement of sporangia away from their previously terminal position and the innovation of indeterminacy. Understanding of the origin(s) of indeterminacy currently rests on comparative analyses of axial development in living bryophytes and vascular plants as the cellular basis of axial elongation in extinct polysporangiophytes is unknown. However, the meristematic activities that generate axial elongation in these two groups are widely disparate. In bryophytes, axial elongation occurs with little cell proliferation (liverworts), by intercalary proliferation beneath sporangia (hornworts) or by embryonic proliferation from an apical cell coupled with later intercalary proliferation (mosses). Given phylogenetic caveats above (see section 4b), the moss proliferative pattern may be the closest living proxy to that of early polysporangiophytes, but apical cell and intercalary proliferative activities in mosses are separated temporally by developmental stage and spatially by sporangium formation (figure 4). In contrast, living vascular plants have shoot apices with juxtaposed stem cell and proliferative activities [73]. The size of the stem cell pool varies between plant groups from a single cell, as in some lycophytes and monilophytes [74,75], through to many in other lycophytes and seed plants [18,76–82], and the coordinated activity of stem cells within the stem cell pool and between the stem cell and subtending zones is required to maintain shoot apex integrity during growth [83]. Thus, comparative development suggests that the displacement of sporangia away from shoot tips and juxtaposition of stem cell and more general proliferative activities were pre-requisites for the origin of indeterminacy [10].

### (b) Genetic pathways for indeterminacy and sporangium development in *Arabidopsis*

There is currently very little experimental evidence of mechanisms involved in the innovation of indeterminate shoot apex functions, but indeterminacy is well characterized in *Arabidopsis*, where two overlying genetic pathways promote cell proliferation and axial elongation. Class I KNOTTED-like homeobox (KNOX) transcription factors are necessary for meristem establishment and maintenance [84,85], acting via cytokinin biosynthesis to promote meristematic cell proliferation [86,87], and WUSCHEL-like homeobox (WOX) transcription factors act in a feedback loop with *CLAVATA* (*CLV*) genes to promote stem cell identity and maintain the size of the multicellular stem cell pool during growth [83]. The genetic basis of sporangium (in angiosperms the pollen sac and nucellus) development is less well understood than mechanisms for indeterminacy [88], but RETINOBLASTOMA cell cycle regulatory proteins suppress WUSCHEL activity to promote entry into germ line specification and meiosis during female germ line development [89], and SPOROCYTELESS MADS-like transcription factors act downstream of the floral organ identity-determining protein AGAMOUS to promote sporogenesis in both male and female germ line development [90,91].

### (c) Genetic bases for the evolution of indeterminacy and sterilization

Meristematic *KNOX* activities are conserved within the vascular plants [92,93], and *KNOX* activity also promotes axial elongation in moss sporophytes [94]. While the activities of *KNOX* genes in liverworts and hornworts are not yet known, these data identify potential homology between mechanisms for intercalary proliferation in bryophytes and apical proliferation in vascular plant meristems (see also [10]). *Physcomitrella* has three globally expressed *WOX13*-like homologues and loss-of-function sporophytes are unable to grow, so conditional or gain-of-function mutants will be required to identify any roles in meristem activity [95] (and also see [96, 97]). *CLAVATA* functions remain unreported or are reportedly absent for non-flowering plants [98,99]. The patterns and position of sporangial development are very variable among non-seed plants (figures 1–3), and the extent to which pathways for sporangial development are conserved among land plants is unknown [100]. *Physcomitrella knox* mutant defects in sporangium development [94,101,102] suggest that *KNOX* genes are upstream regulators of sporangial development in mosses, and provide a potential mechanistic link between sterilization and indeterminacy during shoot evolution [10]. A comparative analysis showed that the transcriptomes of lycophyte, horsetail and flowering plant shoot apices are largely distinct, supporting the ancient divergence time of these lineages and suggesting that the innovation of indeterminate meristem functions may be polyphyletic [59,103,104].

## 6. Stage 3. Leaf evolution

### (a) Lycophyte leaves (lycophylls)

Shoots with leaves first appeared in the fossil record following the innovation of shoots with sterile indeterminate apices, lateral branching systems and lateral sporangia (figures 1 and 3*d–f*). Deep evolutionary divergences within the vascular plant lineage gave rise to today's lycophyte and euphyllophyte flora (figure 1) [11,30,105,106], and living representatives of these lineages all have shoots with leaves (figure 2*e–t*). Early divergences within the lycophyte lineage gave rise to leafless zosterophylls (e.g. *Zosterophyllum*) and lycopsids with partially vascularized leaf-like enations (*Asteroxylon*), with an indeterminate dichotomizing habit (morphologies 8 and 9 in figures 1 and 3*f*,*g*) [9,30]. Both forms are evident in the fossil Rhynie chert flora [17,18,106]. Later lycophyte divergences gave rise to leafy lycopsids (morphology 10 in figure 1), including the extinct order Protolepidodendrales (e.g. *Leclercqia*) (figure 3*h*) and extant groups such as the Lycopodiaceae (clubmoss), sister lineage to Selaginellales (spike mosses) and Isoetalales (quillworts) [11,31]. While living lycophytes are small (figure 2*e–h*), isoetaleans include extinct lycopsid trees such as *Lepidodendron* (figure 3*i*) that were a major component of Carboniferous forests that later fossilized to form coal [107,108].

### (b) Monilophyte fronds

The euphyllophyte stem group included leafless trimerophytes (figure 3*j*,*k*) such as *Trimerophyton*, *Pertica* and *Psilophyton*, which have forking lateral branches with clusters of elongated terminal sporangia [30,38,108], and the euphyllophyte divergence subsequently gave rise to living monilophytes and seed plants (figure 2*h–t*) [38,106,108,109]. The modern monilophyte clade comprises horsetails and ferns (figure 1), and ancient divergences within the fern lineage gave rise to leptosporangiate, marrattioid, ophioglossid and whisk ferns which have widely divergent leaf morphologies (figure 2*i–l*) [109–112]. Living monilophyte lineages are interspersed with extinct relatives (figures 1 and 3*l–n*), and fossil ferns and fern-like shoots have a wide variety of lateral branch arrangements and forms [38,105,106,108]. These are exemplified by *Eospermatopteris* (figure 3*n*), a 3 m tall tree with a crown of spirally arranged flattened first order branches giving rise to multiple dichotomizing branchlets [42], and *Rhacophyton* (figure 3*m*), a 1 m tall plant with partially planar lateral branches and multiple branchlets with some webbing at the distal tips [108,113].

### (c) Seed plant leaves

The modern seed plant lineage (figure 2*n–t*) arose from progymnosperms (figure 3*o*,*p*) such as *Aneurophyton* and *Archaeopteris* (figure 1) [30,108]. *Aneurophyton* has three orders of spiralling lateral branches from which leaves or distinct fertile axes with adaxial sporangia arise, and *Archaeopteris* has planar lateral branching systems with spirally arranged simple leaves or fertile terminal axes (figure 3*p*) [108]. While fossil and phylogenetic data do not fully resolve ancestor–descendant relationships in vascular plant evolution, they demonstrate that lycophytes, monilophytes and seed plants all have leafless fossil precursors and therefore that there were multiple independent origins of vascular plant leaves [105]. Polyphyletic modification of lateral branching systems is considered to have given rise to euphyllophyte leaves in as many as seven to nine independent instances, one in seed plants with the remainder in living and extinct monilophytes [105]. However, the extent of homology in developmental traits such as leaf initiation pattern, determinacy, dorsiventrality and lamination is currently unclear.

### (d) Patterns of leaf development in living vascular plants

Polyphyletic leaf origins are reflected in diverse patterns of leaf development among living vascular plants, reviewed by group in: Ambrose, lycophytes [114]; Tomescu *et al*., horsetails [75]; Schneider and Vasco *et al*., ferns [111,112]; Stevenson, gymnosperms [115]; and Tsukaya, angiosperms [116]. Shared properties of vascular plant leaf development include initiation in a regular phyllotactic pattern at a distance from stem cells that propagate the shoot axis, establishment of proximodistal, mediolateral and dorsiventral axes of symmetry, vein insertion, laminar development, proliferation and growth, but the sequence and extent to which these events occur and are combined vary, leading to diversity in leaf form [105]. The apical functions of different vascular plant lineages are also distinct [76,117,118]. While many vascular plants generate branches subapically (horsetails) [119], on axes at a distance from leaves (some ferns) [111] or in leaf axils (seed plants) [120], lycophytes and other ferns have shoot apices that periodically bifurcate to generate the overall branching form [76–78,114], and a requisite for bifurcation may affect the position of leaf primordia. Patterns of lycophyll development have been identified in a living exemplar of the lycophyte lineage, *Selaginella kraussiana* (figure 2*f*). A clonal analysis showed that two epidermal cells initiate each lycophyll, and that mediolateral cell divisions precede divisions that generate leaf dorsiventrality and tissue layers [77]. However, lycophylls arise from multiple cell layers in Lycopodiaceae (figure 2*e*) and Isoetaceae (figure 2*g*) and patterns of cell proliferation are also divergent among lycophytes [114]. In horsetails, apical cell derivatives divide to attain leaf or intercalary meristem fate [75]. The small leaves (figure 2*h*) have a single vein and emerge in a ring beneath the intercalary proliferative regions that generate the modular shoot axis [75]. Monilophyte fronds (figure 2*i*,*j*) are typified by a shoot-like, tip-down pattern of development with lamina developing by edge-in divisions, and these features may be monilophyte synapomorphies [74,111]. However, there were multiple origins of fronds or leafy forms within the monilophytes and these are reflected in shape diversity [58,112]. Whisk ferns (figure 2*k*) have very small bifid leaves subtending sporangia, ophioglossid ferns (figure 2*l*) have a single entire leaf, and filmy ferns (figure 2*m*) have leaves comprising partially webbed bifurcating axes, with lamina a single cell layer thick [112]. Gymnosperm leaves (figure 2*n–q*) are similarly diverse and range from small and scale-like to large multipinnate forms [115].

### (e) Pathways for leaf development in *Arabidopsis*

Pathways for leaf development are well characterized in flowering plants, exemplified by *Arabidopsis* in which leaves initiate in regular phyllotactic patterns from the peripheral (proliferative) zone of multicellular meristems [121,122]. The position of leaf initiation emerges as an outcome of short-range polar auxin transport principally in the outermost cell layer of the meristem [123]. PIN auxin transporters dynamically direct auxin to maxima on the apical dome, and maximum formation is necessary and sufficient for leaf emergence [64,123–127]. Mechanical forces also contribute to leaf emergence [128], and cell wall loosening by pectin methylesterase or expansin enzymes is sufficient to trigger emergence [129,130]. The recruitment of a pool of meristematic cells into determinate leaf development pathways involves downregulation of meristematic *KNOX* gene activity and maintenance of a *KNOX* off state by ARP transcription factors [84,131,132]. Leaf primordium dorsiventrality is partially inherited from radial symmetries within the shoot axis as primordia emerge for the apical dome [133,134]. *HD-zipIII* genes are expressed centrally in the shoot axis and adaxially within leaves, and *KANADI* and *YABBY* genes are expressed peripherally in the shoot axis and/or abaxially within leaves; loss-of-function mutants respectively generate adaxialized or abaxialized leaves [133,135,136]. *ARP* genes are expressed adaxially, and *Antirrinum arp* mutants also have abaxialized leaves, demonstrating that juxtaposed tissue layers with distinct dorsal and ventral identities are necessary for laminar outgrowth [131,137,138]. Once leaf primordia are established, cell proliferation and growth contribute to leaf shape determination, and many pathways regulating these processes have been identified as an outcome of sophisticated interdisciplinary approaches to understanding how planar forms are attained in plants (e.g. [139]).

### (f) Hypotheses of leaf evolution

The leaf evolution literature has widely recognized lycophylls and euphylls as leaves with distinct evolutionary origins [72,105,106,140–146], and disparity in their size, initiation and venation patterns led to the ‘microphyll’ (lycophyll and horsetail leaves) and ‘megaphyll’ (fern and seed plant fronds and leaves) concepts [73]. The telome theory of leaf evolution proposed that transformative evolutionary processes of unequal branching (overtopping), rearrangement of lateral branches into a single plane (planation) and infilling of spaces between branches with laminar tissue (webbing) generated euphyllophyte leaves [142]. Lycophylls were proposed to have arisen by reduction from a more elaborate precursor state similar to euphyll precursors by a process of evolutionary loss (reduction) [142], as enations by epidermal outgrowth from the stem [46] or by sterilization of lateral branches terminating in sporangia [143]. Zimmermann's hypotheses are dated by the phylogenetic framework and fossil evidence used to infer the direction and nature of character change during leaf evolution [147,148], and more recent literature has moved away from ‘microphyll’ and ‘megaphyll’ terminology as it under-represents the degree of polyphyly in vascular plant leaf evolution [105]. Nevertheless, the telome, enation and sterilization hypotheses highlight developmental processes that may have been generally important in leaf evolution.

### (g) Testing hypotheses of leaf evolution

Evo–devo studies of leaf evolution have only recently started [149,150] and so far have largely focused on leaves with widely disparate origins, for instance comparing lycophyll, fern frond and *Arabidopsis* leaf development pathways [92,151–154]. Analyses of polar auxin transport and/or PIN functions in a lycophyte and a moss suggest that PIN-mediated auxin transport is an ancient and conserved regulator of branch and/or organ position [47,155]. Analyses of HD-ZipIII transcription factor function showed that dorsiventral *HD-zipIII* and *YABBY* expression patterns in leaf initiation are conserved among seed plants, supporting dorsiventrality as a seed plant homology [156,157]. However, *HD-zipIII* activities segregated distinctly among paralogues during gene family evolution, with lycophyte paralogues having functions distinct from seed plant orthologues, and roles for *HD-zipIII*s and *YABBY* in ferns remain to be identified [152,153,157]. An analysis of ARP transcription factor function showed that ARP proteins were independently recruited to suppress *KNOX* activities during leaf initiation in lycophylls and flowering plant leaves [92]. In contrast, KNOX activities are persistent in fern fronds [92,154,158], in line with their late transition to determinate fate [159]. The approaches above support the notion of wide divergence times in vascular plant leaf evolution. Testing more specific hypotheses of character state transition and homology in leaf evolution will necessitate the use of further species in which a particular feature is present or absent and it is possible to do genetics.

### (h) Why have leaves evolved multiple times?

The evidence reviewed above demonstrates that vascular plant leaves have evolved multiple times from branching shoot systems, and that branching forms diversified extensively in lycophyte, monilophyte and seed plant lineages prior to origins of determinate, dorsiventral leaves. Initial constraints to leaf evolution probably involved high atmospheric global temperatures, low stomatal densities and low capacities for water uptake prior to root evolution and the evolution of efficient vascular transport in leaves [16,160,161]. Under these conditions, high incident light absorption would have ‘cooked’ fully webbed leaves or led to vascular embolism in plants' stems [16]. Polyphyletic leaf origins were coupled with declining atmospheric CO_2_ levels, declining global temperatures, increasing stomatal and vein densities in leaves, the evolution of extensive rooting systems and increasing plant competition for space to acquire environmental resources [15–17,162]. In other words, the selection pressures that favour shoots with leaves in today's environment arose at a relatively late stage of plant body plan evolution.

## 7. Conclusion and avenues for future research

### (a) Stages 1 and 2 of shoot and leaf evolution

Combined palaeobotanical, developmental and genetic data are starting to reveal the basis of trait change during polysporangiophyte evolution and pinpoint questions that will reveal a much fuller picture of shoot and leaf evolution if answered. Specific questions for the fossil record include:

— what was the apical organization of Cooksonioid forms,
— are there apical cells,
— what was the cellular basis of bifurcation,
— is there any evidence of intercalary proliferation,
— did vegetative axes develop independently of sporangia, and
— if so, at which evolutionary juncture did such a capacity arise?

By demonstrating the apical activities of polysporangiophytes, answers to these questions will reveal proliferative capacities that predated the origin of indeterminate vascular plant meristems. Fossils of the Rhynie chert could play a key role because they show diversity in relevant traits with high-quality cellular preservation, and occupy key phylogenetic positions.

Developmental and genetic questions include:

— are apical cell and intercalary proliferation coordinated in mosses,
— how do the branching morphologies of *Physcomitrella pinb*, *tel*, *lfy* and *tcp5* mutants arise during development,
— which genes regulate apical cell activity,
— how does apical cell activity cease in bryophytes,
— which genes regulate intercalary proliferation in bryophytes,
— which genes regulate sporangium development,
— are the pathways above conserved among bryophytes, and
— is there conservation between bryophytes and vascular plants?

By answering the above questions, developmental and genetic studies in bryophytes have the potential to reveal mechanisms for apical activity that predated the evolution of polysporangiophytes, i.e. the ancestral mechanisms for axial development, bifurcation and sporangium development. Such mechanisms are likely to have been modified during the radiation of branching lycophyte and euphyllophyte forms.

### (b) Stage 3 of leaf evolution

There are fewer specific questions relating to stage 3 of leaf evolution because the phylogenetic relationships between early diverging lycophyte, monilophyte and seed plant lineages are not well resolved and mutants have not yet revealed phenotypes that are intermediate between living and fossil forms. Analyses of apical, branch and laminar development in early diverging lycophyte, euphyllophyte, monilophyte and seed plant fossils will be required to identify character transitions involved in vascular plant leaf evolution and reveal structural homologies among vascular plant branch and organ systems. While many genes with roles in flowering plant leaf development have been identified, there are few reverse, and no forward genetic data from other vascular plant lineages. The establishment of a fern genetic model [99,163] will go some way to breaking up the wide evolutionary distance between bryophyte [164] and flowering plant [139] models of planar development, but in-depth understanding of leaf evolution and development will require far broader sampling among lycophytes, monilophytes and seed plants [165]. Identifying the developmental and genetic basis of shoot and leaf evolution will be important in future efforts to engineer novel architectural trait combinations to maintain or improve plant productivity in the face of future global change.

## Data accessibility

This article has no additional data.
